# Latent Profile Analysis of Children’s Active Physical Recreation Patterns in Middle Childhood

**DOI:** 10.3390/ijerph22091421

**Published:** 2025-09-11

**Authors:** Stephanie C. Field, John T. Foley, Patti-Jean Naylor, Viviene A. Temple

**Affiliations:** 1 School of Exercise Science, Physical, and Health Education, University of Victoria, Victoria, BC V8P 5C2, Canada; pjnaylor@uvic.ca (P.-J.N.); vtemple@uvic.ca (V.A.T.); 2 Department of Physical Education, State University of New York (SUNY) at Cortland, Cortland, NY 13045, USA; john.foley@cortland.edu

**Keywords:** cluster analysis, social contexts, physical activity, participation, social ecology, motor skills, perceptions of competence

## Abstract

Understanding factors that influence physical activity participation in middle childhood is essential for developing effective interventions. To date, many studies have contributed valuable knowledge on the individual, or person-centered, factors that influence participation, such as motor competence and perceived motor competence. However, there is an increasing body of literature in support of exploring participation through a broader lens, considering additional social ecological factors and their role in participation. Understanding the development of unique combinations of personal and environmental characteristics can shed light on participation patterns over time. Therefore, the aim of this study is to identify clusters of a longitudinal sample of children in grades 2, 3, 4, and 5 (*n* = 155; 55% girls) based on: motor skills; perceived physical competence; active physical recreation; and with whom and where participation occurs. Latent profile analysis results revealed a range of clusters within each grade, with a 3-cluster solution in grade 2, a 5-cluster solution in grade 3, a 4-cluster solution in grade 4, and a 6-cluster solution in grade 5. An analysis of the clusters revealed increasingly diverse clusters over time, with some clusters demonstrating paths toward engagement or disengagement in active physical recreation. The variation in clusters across grades indicates increasing diversity in personal and environmental factors through middle childhood. Recognizing this diversity can allow for teachers, coaches, and instructors to employ instructional styles to accommodate individuals’ differences and maximize participation in a range of physical activity contexts.

## 1. Introduction

Physical activity participation is important to the overall health and well-being of a child [1,2,3]. In order to maximize participation, it is important to understand factors that influence participation in physical activity so appropriate interventions can be implemented [4]. A high volume of research focuses on individual, or person-centered, factors that influence participation, including motor skill proficiency and perceptions of physical competence [5,6,7,8]; however, investigating participation in physical activity through a more holistic lens is being increasingly adopted [9,10]. This can be performed by examining individual and contextual factors concurrently, such as whether children with different levels of motor skill proficiency are participating in different social contexts [9,10]. Additionally, examining participation longitudinally and using time-related factors, such as how often a child is participating in activities, are important pieces of the puzzle [9].

Applying an ecological model to an examination of physical activity participation can provide a multilevel picture of why some children participate and others may not. Högman and colleagues [9] suggest that in an effort to create possibilities for physical activity participation in different settings, “there is a need to consider how influences interact reciprocally with other factors at different levels” [9] (p. 396). Building on the original ecological systems theory [11], Bronfenbrenner and Morris [12] developed a bioecological theory of human development that will serve as a framework for this study. Bronfenbrenner and Morris’ [12] bioecological model has four components that interact to influence development of a number of behaviors: proximal processes, person, context, and time. These components thus comprise the PPCT model, and the proximal processes are “the primary engines of development” [12] (p. 396). Proximal processes are centered around the interactions between the individual, their contexts, and time [12].

‘Person’ refers to individual characteristics such as sex, age, skills, experience, and psychological constructs such as motivation and attitude [9,12]. As indicated in motor development literature, individual characteristics influence participation in physical activity [13,14]. Review evidence strongly supports a positive relationship between motor proficiency and physical activity participation in middle childhood, with the majority of findings based on cross-sectional research [13,15]. Psychological person-level factors such as perceived physical competence and self-esteem also contribute to participation in physical activity [13,14,16]. Perceived physical competence in childhood has been shown to be a predictor of physical activity participation in adolescence [17].

Recent work in this area has made significant progress in our understanding of the roles that actual and perceived motor competence play in shaping children’s physical activity participation. Through the use of latent profile analysis, Barnett and colleagues [8] in Australia demonstrated that children with high actual and perceived competence are more likely to engage in higher levels of moderate-to-vigorous physical activity. Similarly, Lawson and colleagues [18] in England, and Chai and colleagues [19] in China, highlighted the importance of competence-based profiles that explain the role of motivation and engagement in physical activity. This works emphasizes the vital role that these person-level factors (e.g., motor competence and self-perceptions) play in activity participation. As indicated above, there is an increasing emphasis being placed on the influence that ecological contexts have on participation patterns, e.g., [10,20,21]. To date, relatively few studies have explored these factors in combination. More comprehensive approaches that consider person-level factors as well as contextual factors are warranted and would allow for a deeper understanding of the multiple influences on children’s participation patterns.

‘Context,’ as conceptualized in the bioecological model [12], includes the ecological environment in which a child regularly interacts, interpersonal relationships, enjoyment of activities, and the nature of activities in which a child participates (e.g., formal versus informal) [9]. A child’s participation in physical activity can be influenced by a number of contextual factors including parental behavior, school physical education programs, afterschool and camp programs, built environments, and access to physical activity resources [10]. Children who regularly participate in physical activity tend to have strong parental influence, friends who also participate and believe active physical recreation is fun, attend schools that encourage active physical recreation, and participate in school and community-based active physical recreation programs [10]. In contrast, social relationships, such as peer interactions at school, can also decrease participation in physical activity if a child feels they may be ridiculed or judged while participating [22].

The final component of the PPCT model is ‘time,’ which can be thought of as chronological time as well as time spent interacting [12]. It is recommended that children between the ages of 5–17 years of age engage in a minimum of 60 min of moderate-to-vigorous physical activity per day in order to achieve associated benefits [23]. For many Canadian children, however, this is not the case, with only 39% of 5- to 17-year-olds meeting the recommended guideline [24]. This is concerning as physical activity levels in childhood are indicative of physical activity in adulthood [25]. As children move through middle childhood, participation in physical activity begins to compete with other activities such as household chores and school-related tasks (e.g., homework) [26].

It is possible, and likely, that there are a number of unique combinations of behaviors and characteristics exhibited by children who participate in regular physical activity and those who do not [8,18,27]. Therefore, the aim of this study was to identify variation in combinations (i.e., clusters) of person, context, and time characteristics of children in grades 2, 3, 4, and 5. These clusters were based on patterns of motor skill levels, accuracy of perceived physical competence, active physical recreation, and with whom and where active physical recreation occurs. To accomplish this aim, the following research question was addressed: What are the clusters of children in grades 2, 3, 4 and 5, based on levels of motor skills, accuracy of perceived physical competence, active physical recreation participation, and with whom and where participation occurs?

## 2. Materials and Methods

A sequential design was used to examine clusters of children in grades 2, 3, 4, and 5. Approval for this study was granted by the University of Victoria Human Research Ethics Board (protocol number 10-246) and School District #61 in Victoria, British Columbia, Canada. Data were collected as part of a larger Motor Development Study that took place from 2010–2017.

### 2.1. Participants

All grade 2 students attending one of eight participating schools during the 2012-13 (cohort 1) and 2013-14 (cohort 2) school years were provided with an invitation to participate. Parents provided written consent and children provided written assent at the beginning of each data collection year. Children who consented annually were tracked in grades 2, 3, 4, and 5.

Children were eligible to participate in this study if they had complete data for motor skills, perceptions of physical competence, and active physical recreation for grades 2, 3, 4, and 5. Participants were *n* = 155 boys and girls (55% female; Mean age = 7.8 years).

### 2.2. Measures

Fundamental motor skills (FMS) were assessed using the Test of Gross Motor Development 2nd ed. (TGMD-2) [28] and perceptions of physical competence using the Pictorial Scale of Perceived Competence and Social Acceptance for Young Children [29] for grade 2 participants and the Self-Perception Profile for Children [30] for participants in grades 3, 4, and 5. The use of two perceived competence scales was selected for this study to match the appropriate developmental level of participants. The Pictorial Scale is intended for use by “first- and second-graders” [29], while the Self-Perception Profile is to be used by children in grades 3–8 [30]. Each scale contains a developmentally appropriate format for participants. The Pictorial Scale includes images that correspond to each question, allowing for greater comprehension by younger participants [29]. Questions in the Pictorial Scale are framed using concrete language suitable for younger children (e.g., “running fast”), versus more abstract language (e.g., “athletic”) used in the Self-Perception Profile.

Motor skills and perceptions of physical competence scores from these assessments were subsequently used to generate accuracy *z*-scores. Additional information about these measures and accompanying procedures, including validity and reliability, are fully detailed in Field et al. [31]. Active physical recreation was measured using the Children’s Assessment of Participation and Enjoyment (CAPE) [32].

#### 2.2.1. Fundamental Motor Skills and Perceptions of Competence

The TGMD-2 is a developmental test of fundamental motor skills consisting of 12-items and designed as both a norm- and criterion-referenced assessment. The test is organized into two distinct sub-scales: (1) locomotor skills (run, gallop, slide, hop, jump, and leap); (2) object control skills (dribble, catch, strike, throw, roll, and kick). Each of the 12 test items is assessed independently and consists of 3–5 components. For example, when catching, one component is “arms extend while reaching for the ball as it arrives” [28] (p. 36). All components are scored dichotomously. When a child performs a skill component according to the TGMD-2 performance criteria, they receive a ‘1’. If the component criteria is not met, a ‘0’ is awarded. The total possible raw score range for this assessment is 0–96. The raw score range for each subscale is 0–48. 

As mentioned, two measures for self-perceptions were used in this study: The Pictorial Scale of Perceived Competence for Grade 2 participants [29], and the Self-Perception Profile for Children for Grade 3–5 participants [30]. These self-report measures contain subscales relevant to different developmental domains (e.g., social, cognitive, etc.). For the purposes of this study, the physical domain subscale was used. The Pictorial Scale (Grade 2) refers to this perception subscale as ‘physical’, while the Self-Perception scale (Grades 3–5) refers to it as ‘athletic’, so as not to be confused with ‘physical appearance’ subscale present in the Self-Perception scale. For the remainder of this study, we will refer to both physical (Grade 2) and athletic (Grades 3–5) subscales as ‘perceptions of physical competence’. There are six items (i.e., questions) in the perceptions of physical competence subscale. Each item contains a negative and positive statement regarding proficiency of a task. For example, a positive statement such as “Some kids do very well at all kinds of sports” [30] (p. 6) suggests proficiency in sports, while the negative statement, “Other kids don’t feel that they are very good when it comes to sports” (p. 6), indicates a lack of proficiency in sports. Children are asked to select which statement they most identify with; that is, which is ‘most like them’. They are then further asked to identify if their selected statement is ‘really true’ or ‘sort of true’. The score range for each item is 1–4. Lower scores represent the most negative selection (e.g., a negative statement that is ‘really true’), and higher scores represent the most positive selection (e.g., a positive statement that is ‘really true’).

#### 2.2.2. Active Physical Recreation

The CAPE was created to assess children’s and youth’s (6–21 years of age) voluntary participation in formal and informal recreation and leisure outside of mandated school curricula [32]. Five dimensions of participation are assessed by the 55-item CAPE survey: (a) diversity, the number of activities in which a child participates, (b) intensity, how often the child participates in an activity, (c) with whom the child participates, (d) where the child participates, and (e) enjoyment of the activities. Enjoyment of activities was not included in this study. The CAPE is sub-divided into nine activity categories, two of which are Organized Sports and Active Physical Recreation [21]. Combined, these two categories include 17 activities (e.g., swimming, team sports, individual physical activities).

The diversity dimension of the CAPE is recorded as a binary score. Children are awarded a ‘1’ if they report participating in an activity (e.g., they have gone for a hike in the previous four months). Subsequently, they provide responses for the remaining dimensions associated with that activity (e.g., how often they hike, with whom they hike, etc.). If they have not participated in the activity in the previous four month, the administrator records a ‘0’ and moves onto the next activity. The Intensity dimension represents how often a child has participated in an activity. Scores for Intensity range from ‘1’ (once in the previous four months) to ‘7’ (everyday); a score of ‘0’ is recorded if the child did not do the activity in the previous four months. The dimensions of ‘With Whom’ and ‘Where’ represent the type of person a child does activities with (e.g., alone, with friends, with family), and where they participate (e.g., at home, in the neighborhood, in the community). The scores for these dimensions range from 1–5 and 1–6, respectively, with the lower scores indicating participation in the most immediate or closest context (e.g., alone, at home), and higher scores representing broader contexts (e.g., with others, beyond your community).

Content validity for the CAPE was established through a thorough literature review on participation, expert review, and pilot testing [32], and construct validity has been examined in terms of the expected relationships between participation and environmental, family, and child characteristics such as sex and motor skill proficiency [33,34]. Construct validity of the CAPE has also been examined by Temple et al. [34] among 74 children in their first year of school. The mean test–retest reliability correlation coefficient for diversity is 0.75, ranging from 0.67 for skill-based activities to 0.78 for formal activities [32].

### 2.3. Procedures

Research assistants, trained annually by the project coordinator and lead investigator, collected data in adherence will administration manuals for each measure [28,29,30,32].

Fundamental motor skills were assessed over two, 30-min physical education classes. Participants were invited into the gymnasium and organized into four groups. In each session, children rotated through four motor skills stations, completing all items in the TGMD-2. All children, both consented and not-consented, participated in the motor skills stations; however, only those who consented to participate were video recorded (Sony Handycam hdr-cx240). The videos were subsequently downloaded onto a secure server at the University of Victoria and assessed by a research assistant trained in TGMD-2 scoring procedures [28].

Depending on the school and teacher schedules, administration of the questionnaires was completed either immediately following the motor skills stations, or on occasion, within one or two days following the motor skills assessment. Both questionnaires (perceptions of physical competence and the CAPE) where completed in one sitting, taking an average of 20–40 min depending on the child. Questionaries were administered during school hours by a trained research assistant in a quiet setting (e.g., school library, multi-purpose room) to reduce distraction. At the outset of each questionnaire, participants were reminded that there were no right or wrong answers, and to respond however they felt was true for them. Some Grade 3, 4, and 5 participants wished to complete the perceptions of physical competence questionnaire independently. After administration of the first few questions by a trained research assistant, the administrator remained seated with the child to ensure correct completion of the questionnaire.

To ensure accurate reporting of activity participation per the CAPE administration manual, children were reminded to only respond for activities they had participated in outside of class time, voluntarily, and within the previous four months [32]. The CAPE picture binder, which includes one image per activity, was provided to participants at the outset of the questionnaire administration to assist in children’s understanding [32]. Children followed along with the picture binder while the research assistant administered the questionnaire.

### 2.4. Data Treatment and Analysis

It is important to use statistical techniques that allow for the discovery of unique groupings, or clusters, of behavioral patterns and characteristics in order to provide a comprehensive picture of the combination of person, context, and time components exhibited by different children. Understanding the unique clusters of children may help tailor active physical recreation and education programs for children who exhibit a unique combination of factors. For this reason, this study employed latent profile analysis to identify clusters of children from observed data [35,36]. Latent profile analysis is a person-centered statistical technique that uses a multidimensional perspective to empirically identify clusters, based on responses to multiple items of interest (e.g., active physical recreation, motor competence, perceived competence) [36,37]. This identification will help to increase the effectiveness of health behavior interventions by informing the tailoring of individually adapted interventions aimed at increasing physical activity participation [38]. Although there is no formal consensus on the sample size required to effectively use latent profile analysis [39], a minimum sample size of *N* = 150–200 is recommended [40]. The latent profile analysis for this study was conducted using STATA (version 15).

The analysis was used to generate clusters of children in grades 2, 3, 4, and 5 based on participants’ motor skills, accuracy of perceived physical competence, active physical recreation participation, and with whom and where participation occurs. Active physical recreation values were calculated using the 17 organized sport and active physical recreation activities in the CAPE, and accuracy *z*-scores were generated using motor skills and perceived physical competence raw scores. In Stodden and colleagues’ [14] original model, spirals of engagement and disengagement are associated with high and low skill and perceptions of competence levels, respectively. However, as demonstrated in our previous work [31], it is not solely the levels of perceptions of competence, but also accuracy of those perceptions that is connected to participation; therefore, accuracy, rather than perceptions of competence, was used in this study.

Total (object control and locomotor skills) raw motor skill scores were used in the latent profile analysis. Additionally, percentage of maximum possible (POMP) scores were calculated for TGMD-2 scores to help in the interpretation of the clusters. The following formula for POMP was applied: [(observed score − minimum possible)/(maximum possible − minimum possible) × 100] [41]. *Z*-scores, rather than raw scores, were used to create figures, as *z*-scores allow for better visual comparison when examining clusters. Converting raw scores into *z*-scores allows for direct comparison between variables of different clusters. Accuracy was already represented as a z-score; however, motor skills, active physical recreation, with whom, and where were converted to *z*-scores to facilitate these visual comparisons.

A series of nine models were run per grade (2-class to 10-class in each grade) using the input variables listed above (e.g., active physical recreation, accuracy, etc.), with the understanding that additional models may have been required; however, 9 models were sufficient in each grade. Although latent profile analysis allows for modeling with covariates, when using sex as a covariate, the model consistently collapsed after four iterations as sex was represented as a binary variable. This precluded a final best fit model with sex as a covariate. Sex was removed from the cluster analysis and, instead, the proportion of boys and girls in each cluster was reported descriptively. Schwarz’s Bayesian information criteria (BIC) and Akaike’s information criteria (AIC) were selected as best fit indices to look for parsimony within the data [36]. After running the models, three researchers compared the fit data using BIC and AIC values to determine the most appropriate model (e.g., number of clusters) for each grade. Additionally, scree plots using AIC and BIC values were generated for each grade in Microsoft Excel (version 16) to identify the point of inflection for each value set, which is the suggested point of cut-off [42]. In keeping with latent profile analysis interpretation, the BIC values were used as a primary fit and AIC was used as a supplementary value to further support the selection of the best fit model [36]. The model with the smallest value of both BIC and AIC was considered to be the best fit. Profiling, the process of generating a description of the clusters with reference to the input variables, was then completed for each cluster.

## 3. Results

**Figure 1:**
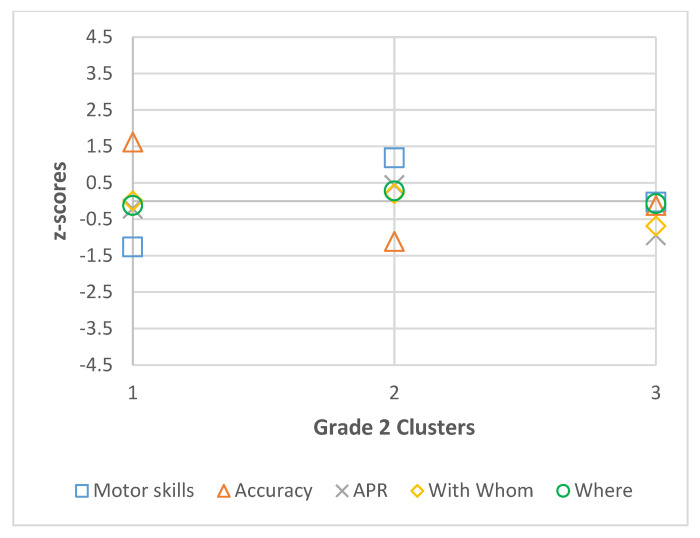
Grade 2 Cluster z-scores for Motor Skills, Accuracy, Active Physical Recreation (APR), With Whom, and Where.

**Figure 2:**
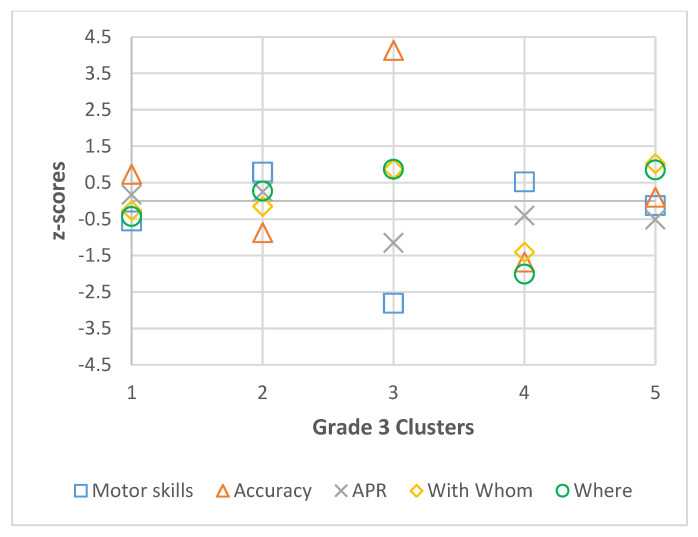
Grade 3 Cluster z-scores for Motor Skills, Accuracy, Active Physical Recreation (APR), With Whom, and Where.

**Figure 3:**
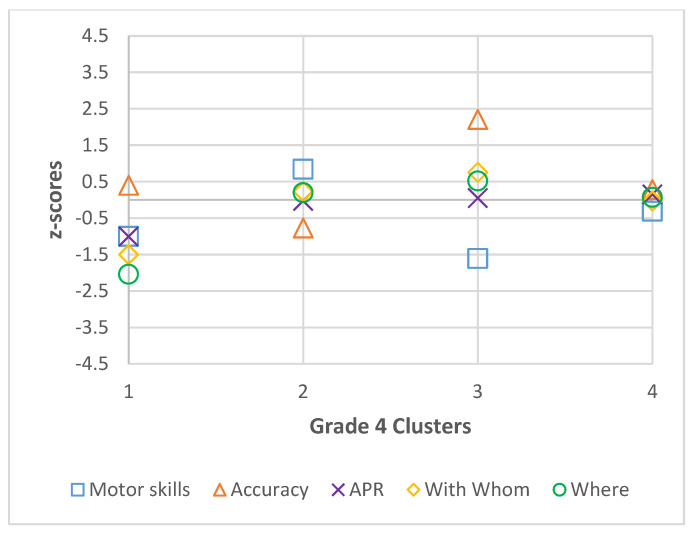
Grade 4 Cluster z-scores for Motor Skills, Accuracy, Active Physical Recreation (APR), With Whom, and Where.

**Figure 4:**
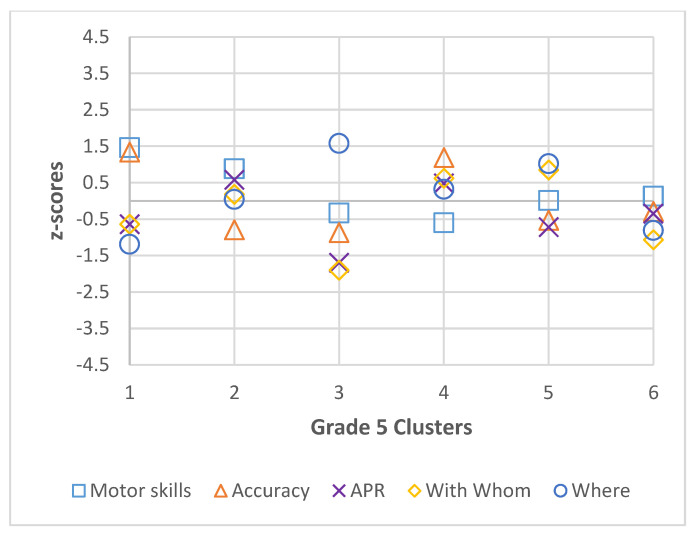
Grade 5 Cluster z-scores for Motor Skills, Accuracy, Active Physical Recreation (APR), With Whom, and Where.

Descriptive statistics (mean and standard deviation) were calculated by grade for all participants for motor skills, accuracy, active physical recreation, with whom, and where, and are presented in Table 1, Table 2, Table 3 and Table 4. The best fit models for each grade, including coefficient values (Coef.) and 95% confidence intervals (CI) of the latent profile analysis are also presented in Table 1, Table 2, Table 3 and Table 4 and visual representations of standardized coefficient values are presented in Figure 1, Figure 2, Figure 3 and Figure 4. In grade 2, fit data indicated a final 3-cluster solution (BIC = 2585.641; AIC = 2518.686). Grade 3 latent profile analysis results indicated a 5-cluster solution (BIC = 2623.623; AIC = 2520.147). In grade 4, the best fit was a 4-cluster solution (BIC = 2562.866; AIC = 2477.65). Lastly, in grade 5, BIC (2599.181) and AIC (2477.444) values revealed a 6-cluster solution to be the best fit. In each grade, all variables significantly contributed to the clusters (significance level set at *p* < 0.05).

## 4. Discussion

The aim of this study was to identify variation in patterns of person, context, and time characteristics of children in grades 2, 3, 4, and 5 based on their motor skills, accuracy of perceived physical competence, active physical recreation, and with whom and where active physical recreation occurs. Examination of how these variables congregated highlighted the diversity of children’s needs that must be considered when designing and delivering programs and learning experiences. The results revealed a general trend of an increasing number of clusters in the higher grades; with a 3-cluster solution in grade 2, a 5-cluster solution in grade 3, a 4-cluster solution in grade 4, and a 6-cluster solution in grade 5. The variation in the number of clusters across grades is indicative of increased variation in children’s abilities, self-appraisals, what children are doing, and with whom and where they are doing it.

In this discussion, we examine patterns of clusters both within and between grades. It is important to be mindful when comparing clusters that score interpretations (e.g., low, high, narrow, connected) are relative to other clusters and not to normative results or other findings, with the exception of using POMP scores to aid in the description. Additionally, although participants in this study are part of a longitudinal sample, cluster reclassification is occurring in each grade; therefore, it is possible children who were in one cluster (e.g., Cluster 1) in one grade could be in another cluster (e.g., Cluster 2) in a subsequent grade. The changing proportions within the clusters confirm the presence of this reclassification. As the aim of this study is centered around active physical recreation participation, the following cluster selections have been driven by the active physical recreation scores in each cluster. 

Overall, our analysis revealed a number of distinct profiles that align with fundamental models of engagement and disengagement [13,14]. Although data collection for participants was completed in 2017, the developmental processes captured in our analysis continue to be central to discussion of participation, motor development, and physical literacy. Further, the addition of social ecological factors such as ‘with whom’ and ‘where’ in our latent profile analysis extend previous work in this area that have used cluster analysis to explore person- or individual-level factors, e.g., [8,18,43,44,45]. Recent evidence confirms that children’s activity patterns are shaped not only by motor skills and perceptions of competence, but also by ecological influences such as peers, parents, and teachers, e.g., [20,21]. Few studies have considered this unique combination of variables in latent profile analysis, and longitudinal work in this area is limited. We hope our evaluation of cluster patterns over time can provide a comprehensive benchmark against which future work can be compared.

### 4.1. The ‘Clusters 1’: Low, Low, Over, and Narrow

There were several distinct within- and across-grade consistencies in the cluster formations. In each grade, there was a cluster of children (named Cluster 1) who seemed to be on a path toward a spiral of disengagement [14] from active physical recreation. Children in Cluster 1 in each grade had low motor skills, low participation rates, and inaccurate perceptions, with a tendency to overestimate their abilities. Although data collection for this study completed in 2017, low physical activity levels and low motor skill levels remain pressing issues for Canadian children. The 2024 ParticipACTION Report Card indicates that only 39% of Canadian children are achieving the recommended guideline of 60 min of daily moderate-to-vigorous physical activity. Further, motor skills levels in this study are supported by recent Canadian findings of grade 2 and 3 children as assessed by the Physical Literacy Assessment for Youth (PLAY) tools; specifically, the PLAYfun tool assesses children’s performance in 18 fundamental movement tasks [46]. Findings from Tang and colleagues [45] are consistent with pre-COVID-19, and COVID-19 era motor skills levels, with children demonstrating movements associated within the ‘emerging’ stage of the PLAYfun 4-category scale (initial, emerging, acquired, proficient). Skills at this level represent movement patterns that are characterized as having a “limited number of major gaps, but able to execute basic sequencing of the task” [47] (p. 6). Additionally, these children participated in more narrow social contexts than children in the other clusters. In each grade, the proportion of girls in Cluster 1 was higher than that of boys, with girls comprising between 62% and 82% of the cluster. As Cluster 1 is partially characterized by low motor skill levels, the distribution of boys and girls may be partly explained by the low skill levels of girls in this sample. Descriptive statistics presented in our previous work using the same sample of children as the present study [31], indicate that girls have lower skill scores than do boys. Of primary concern for Cluster 1 children are the low motor competence and inaccurately high perception levels of these children, as these are important predictors of activity participation [13,17,48].

Relatively narrow social networks are also a characteristic of Cluster 1, especially in grades 4 and 5. Coefficient values for Cluster 1 in grades 4 and 5 indicate that children in these clusters spend their active physical recreation time with their family and in narrow social environments. The ‘where’ coefficient values in Cluster 1 in grades 4 and 5 correspond to at a relative’s home, although, as revealed in our previous work, minimal active physical recreation took place in that context. Instead, these coefficient values are likely the mean representation between participation occurring at home and in your neighborhood. Regarding social involvement for those in the low cluster, establishing positive relationships with parents, coaches, teachers, and peers can be a key motivational tool for physical activity participation [49]. Strategies for building positive relationships in active physical recreation contexts will be discussed later in this section.

### 4.2. The ‘Clusters 2’: High, High, Under, and Connected

There was also a consistent cluster of children, named Cluster 2, who appeared to be in a spiral of engagement [14]. Children in Cluster 2 had relatively high motor skill proficiency, high participation rates, and participated in more distal social contexts. But, similar to the low cluster, children in Cluster 2 also had inaccurate perceptions, with a tendency to underestimate their abilities. Although children in this cluster appeared to be in a cycle of engagement as evidenced by their relatively high active physical recreation scores, our previous work indicates that underestimators have lower participation rates and less skill development relative to their average and overestimating peers [31]. Therefore, it would benefit these children to increase their perception levels to more accurately match their skill level. Strategies for development of accurate perceptions are discussed below. It should be noted that determining which cluster should be labeled as ‘Cluster 2,’ was challenging for grade 4. The differences between active physical recreation scores were negligible between two clusters in grade 4 (cluster 2 and cluster 4); therefore, we examined other differences and found motor skills emerged as a differentiating variable. As such, the cluster that had both high active physical recreation and the highest motor skills scores was selected as Cluster 2 in grade 4.

### 4.3. Clusters 3, 4, 5, and 6

The remaining clusters (3, 4, 5, and 6) had varied composition and less consistent patterns across the grades than clusters 1 and 2. From grade 2 to grade 5, the number of clusters doubled. The three clusters in grade 2 were relatively tidy, with approximately one-quarter of participants in Cluster 1 (low, low, over, and narrow), one-quarter in Cluster 2 (high, high, under, and connected), and roughly half of the participants in the remaining cluster (Cluster 3). By grade 5, however, the six clusters, particularly clusters 3, 4, 5, and 6, revealed a more complex picture with different cluster combinations emerging from the data, and cluster membership that ranged from approximately 3% to 32% of participants. The increase in the number of clusters is indicative of greater variation in children’s responses to the continuous input variables (e.g., with whom, where, participation). Although speculative, a partial explanation for increased cluster numbers may include children having different opportunities for participation, competing interests in other activities (e.g., music), or priorities that inhibit participation in active physical recreation (e.g., responsibilities at home). The reason for increased cluster numbers was not measured in this study; however, the cluster numbers do reinforce that, particularly toward the end of the middle childhood, children are becoming more diverse in their active physical recreation participation patterns. As participation and skill development are cyclically related [14], this suggestion is consistent with evidence that environmental affordances such as practice and experience lead to greater differentiation in motor ability with age [50]. Future research should examine participation patterns in multiple CAPE activity categories (e.g., social activities, quiet recreation activities, self-improvement activities) to help identify if children are moving toward participation in other areas.

The proportion of children in each cluster also fluctuated across the grades. Encouragingly, the proportion of children who presented with relatively high motor skills, active physical recreation levels, and social contexts (Cluster 2), increased from 19% of participants in grade 2 to 32% in grade 5. This indicates that some participants are reclassified from clusters that are considered more at risk for active physical recreation disengagement (e.g., Clusters 1) into the ‘high’ cluster as they move through the grades.

There were a number of small clusters (≤4%) of children. Cluster 3 in grade 3 is particularly unique. The two children in this cluster, one boy and one girl, had extremely poor motor skills, wildly overestimated their abilities, had low participation rates, and yet participated in distal environments with their family. As the children in this cluster had noticeably inaccurate perceptions which were represented by high *z*-scores, it was possible to trace the individual active physical recreation CAPE item responses for these two children. In particular, we found that these children participated in activities that required skills not assessed by the TGMD-2; for example, each of the children swam, rode bikes, and played on equipment (e.g., climbing) in distal environments. This cluster, as with the other small clusters, give pause for the following methodological considerations and limitations. It is also important to note that although our analysis of the smaller clusters was a catalyst for recognizing and highlighting the following limitations, interpretation of all clusters should take these limitations into consideration.

Firstly, the CAPE measures 17 active physical recreation items; however, it is not known if children were participating in physical activities not measured by the CAPE. Secondly, during childhood “skill specificity in task performance seems clearer with increasing age” [50] (p. 222). Although the TGMD-2 was designed to measure general motor abilities [28], it does assess 12 specific locomotor and object control skills, which may not be sensitive to skills associated with some motor tasks, such as swimming and riding bikes. Although some clusters demonstrate very low levels of skill proficiency, and overall the sample has low levels, there may be some children who have a greater generalized motor proficiency than is measured here. In order to maximize participation, it is necessary for children to become proficient in fundamental motor skills as these ‘building block’ skills contain general movement patterns that can be transferred between contexts (e.g., overhand throw movement can be used in a tennis serve or overhead badminton clear) [51] and would allow more opportunities for engagement. Motor development and physical activity experts have put forth a recommendation for new terminology to include not only locomotor and object control skills, but movement patterns such as swimming and cycling, that are necessary for engagement in a number of physical activities [52]. Future research would benefit from measuring an increasing variety of movement patterns to gain a better understanding of a child’s overall movement proficiency. Additionally, it is important to recognize a further limitation of the CAPE being that it only records the most distal social interactions a child has experienced during activity participation in the previous four months. For example, participating in an activity alone is awarded a score of ‘1’, while participating in an activity with friends is awarded a ‘4’. It is possible a child may have participated in an activity such as playing on the playground both alone and with friends. In such a case, only a score of ‘4’ is recorded. Similarly, locations that are more proximal (e.g., at home), are awarded a lower score than those more distal (e.g., in your neighborhood, at school). For the purposes of this study, capturing and reporting the most distal social context was appropriate. While the CAPE remains a valid and reliable tool to capture activity participation in various social contexts [32], it is necessary to acknowledge these limitations. In future, it is recommended that measures of social contexts and activity participation capture the full range of environments for any given activity, to further understand the complexity of participation patterns in middle childhood.

An examination of cluster membership was approached through the lens of the PPCT model [12]. Although this study did not test the PPCT model per se, the interactions between individual (e.g., motor skills, accuracy) and environmental factors (e.g., social contexts) were considered. As indicated, Clusters 1 and 2 had patterns of consistent interaction between individual and environmental factors across the grades, while clusters 3 through 6 had much more varied interactions. Particularly in grade 2, there was a clear pattern between individual and environmental factors, although it was one of no interaction. From grade 3 onward, however, with whom and where became more differentiated between the clusters and, with the exception of Clusters 1 and 2, there were fewer clear interactions between individual and environmental factors in the later years. A possible explanation for these patterns may be found by examining the interactions from early to middle childhood to see when a shift in interaction between individual and environmental factors takes place. Regarding changes in with whom children spend their time during middle childhood, this may be partly explained by an expected shift toward more time spent with friends. As children move toward adolescence and move along the parent-to-peer pathway [53], they may experience increasingly stronger interactions between individual and environmental factors. It may be that the middle childhood years are a time of relative stability in regard to individual and environmental interactions.

#### Approaches to Facilitate Individual Development in Diverse Environments

Our findings highlight that throughout middle childhood, children have increasingly diverse participation backgrounds and personal resources (e.g., motor competence). To optimize children’s development, instructional approaches need to accommodate this diversity. Two related instructional approaches that will be helpful when instructing children in the middle years are differentiated instruction [54] and the inclusion style of teaching [55]. Differentiated instruction is a pedagogical approach designed to meet the needs of individual children within a group context (e.g., physical education, camp, and sports programs) [54]. Paramount in differentiated instruction is learning about each child. An important first step to achieve this is to conduct a needs assessment prior to, or at the beginning of, a program, class, or practice season [54]. This involves “…taking into account what each child needs from this climate in order to feel comfortable, motivated, and successful” [54] (p. 19). Both children and parents can contribute to the discovery of the child’s areas of interest, feelings toward an activity, goals, and/or skill level. In terms of the bioecological model, this is about finding out about the ‘person’ (i.e., skills, attitude, motivation, past experiences, etc.) [12]. This information can then be useful to provide a tailored learning environment where each child can succeed. Once we learn about a child’s individual needs, instructors are better equipped to construct learning environments that cater to varied interests and strengths.

As previously mentioned, the cluster analysis results revealed considerable diversity in both motor skill levels and accuracy of perceptions. Participants with the highest motor skills consistently underestimated their abilities, while those with the lowest motor skills overestimated. These contrasting clusters require assistance in developing in different areas, which presents a challenge to the instructor, coach, or teacher who may have children with these differing profiles in one context (e.g., classroom, team sports). Instructional approaches that protect children’s self-concept while encouraging the development of their motor competence will be helpful for children in Clusters 1 (overestimators with low skills). Harter [16] argues that “…promoting more realistic self-evaluations among overraters [sic] should be a first step” (p. 264) of interventions to promote skill development. This, she suggests, is because children who overestimate their abilities tend to select ‘easier’ activities that ensure success in an effort to protect confidence. As a result, children may choose activities that are not especially challenging, and thus, do not develop their skills optimally. Yet, at the same time, their inflated perceptions are reinforced. Promoting more realistic self-evaluations for overestimators may be an appropriate recommendation based on cluster findings from this study.

We would, however, be remiss not to consider findings of our previous work [31] in this discussion; that is, to consider that children in grades 3–5 who overestimated their motor skills, also demonstrated the most motor skill improvement of their peers. While the cluster analysis provided evidence that overestimation is associated with poor motor skill levels, the trajectory of skill development for this group from grade 3–5 suggests that overestimating is associated with some benefits in middle childhood. Although we advocate that promoting realistic self-evaluations is important for overestimators, it may not necessarily be the ‘first step’ when intervening to promote skill development for this group. As Albert Bandura stated, if people did not err toward overestimation in their self-appraisals, then their self-efficacy beliefs will only reflect what they can do, and they may “not mount the extra effort needed to surpass their ordinary performance” [56] (p. 343). For underestimators, we suggest the promotion of realistic self-appraisals should be prioritized as we have found that underestimators demonstrated the least skill improvement across grades and had lower participation rates than their average and overestimating peers [31]. Coaches and instructors can help underestimators recognize their actual skill level by helping a child identify and affirm their actual abilities. Addressing inaccuracy for this group may be more complex. As Harter [16] mentions, underestimating at an early age may be reflective of larger problems (e.g., emotional abuse) and may require a more profound examination (e.g., personal counseling) to address the root cause of underestimation.

The inclusion style of teaching [55] is one approach that instructors, coaches, and teachers may find particularly useful to operationalize differentiated instruction as it can be used to reduce the fear of failure and allow children to try out the interplay between self-aspiration and reality. This teaching style is characterized by providing learners with multiple options of the same task with varying degrees of difficulty. For example, when teaching a child to juggle, the instructor may provide options of the type of equipment that can be used (e.g., scarves, beanbags, or pins). The learner is then tasked with performing a self-assessment and selecting one of the choices as an entry point, for example, the beanbags. After an attempt with the initial selection, the learner assesses their performance against criteria provided by the instructor and makes a choice: to continue to use the bean bags, to select the scarves (an easier challenge), or to select the pins (a more difficult challenge). “The objective is to teach the learner to make appropriate decisions about which level … [they are] most capable of performing” [55] (p. 162). This selection process has important physical, social, cognitive, and emotional implications. Physically, although not the primary objective of this style, a child may work toward skill mastery by selecting an optimally challenging skill. Socially, the inclusion style helps children learn to accept individual differences; and, cognitively, to develop more accurate perceptions through increased actual competence and internal information (e.g., ease of learning with a particular object). Emotionally, this style engages an aspect of a child’s self-concept (i.e., perceptions of competence) as they are required to perform a self-evaluation and decide on what they feel is an appropriate level of challenge. As Mosston and Ashworth [55] state, “…it is almost like a bargaining session with oneself” (p. 159). This interplay between self-aspirations and reality is a key feature of Mosston and Ashworth’s [55] inclusion style of teaching, and may offer support for children as they learn to evaluate both their perceived and actual abilities while participating in diverse social contexts.

## 5. Conclusions

In this study, latent profile analysis was used to generate clusters of children in middle childhood based on motor skills, accuracy of perceptions, active physical recreation participation, and with whom and where that participation occurred. Latent profile analysis revealed a range of clusters across the grades, with an increasing number of clusters from grade 2 to grade 5, reflecting greater variation in skills, perceptions, participation, and the social contexts where participation occurred. Clusters of children with ‘low, low, over, and narrow’ and ‘high, high, under, and distal’ scores were consistent in each grade, suggesting some children are at risk of disengagement from active recreation while others are on a path to continued participation.

## Figures and Tables

**Table 1:** Grade 2 Descriptive Statistics and Latent Profile Analysis Coefficient and Confidence Interval Results.

	All Participants (*n* = 155)		Cluster 1 (*n* = 29, 62% Girls)		Cluster 2 (*n* = 29, 41% Girls)		Cluster 3 (*n* = 97, 57% Girls)	
Variable	M	*SD*	Coef.	95% CI	Coef.	95% CI	Coef.	95% CI
Motor skills	60.50	8.47	49.84	[46.83, 52.84]	70.59	[67.18, 73.99]	60.24	[57.93, 62.58]
Accuracy	−1.0 × 10^−3^	1.23	1.62	[1.18, 2.07]	−1.11	[−1.56, −0.67]	−0.12	[−0.44, −0.21]
APR	1.90	0.78	1.74	[1.41, 2.06]	2.24	[1.89, 2.59]	1.83	[1.64, 2.01]
With whom	2.94	0.54	2.94	[2.70, 3.18]	3.01	[2.82, 3.29]	2.90	[2.77, 3.03]
Where	3.80	0.72	3.72	[3.42, 4.03]	4.01	[3.73, 4.29]	3.76	[3.59, 3.93]

**Table 2:** Grade 3 Descriptive Statistics and Latent Profile Analysis Coefficient and Confidence Interval Results.

	All Participants (*n* = 155)		Cluster 1 (*n* = 62, 63% Girls)		Cluster 2 (*n* = 51, 41% Girls)		Cluster 3 (*n* = 2, 50% Girls)		Cluster 4 (*n* = 7, 71% Girls)		Cluster 5 (*n* = 33, 58% Girls)	
Variable	M	*SD*	Coef.	95% CI	Coef.	95% CI	Coef.	95% CI	Coef.	95% CI	Coef.	95% CI
Motor skills	64.46	9.33	59.31	[57.05, 61.56]	71.84	[68.54, 75.14]	38.20	[28.43, 47.97]	69.34	[63.80, 74.89]	63.27	[59.98, 66.56]
Accuracy	−5.0 × 10^−4^	1.22	0.73	[0.47, 1.00]	−0.87	[−1.21, −0.53]	4.13	[2.96, 5.31]	−1.68	[−2.35, −1.02]	0.10	[−0.41, 0.62]
APR	1.85	0.74	1.97	[1.78, 2.16]	2.03	[1.78, 2.29]	1.00	[0.04, 1.96]	1.55	[1.05, 2.06]	1.47	[1.15, 1.79]
With whom	3.03	0.62	2.87	[2.71, 3.03]	2.94	[2.74, 3.14]	3.57	[2.90, 4.24]	2.16	[1.78, 2.53]	3.66	[3.39, 3.94]
Where	3.85	0.69	3.55	[3.38, 3.73]	4.03	[3.86, 4.21]	4.45	[3.76, 5.14]	2.46	[2.01, 2.91]	4.44	[4.20, 4.68]

**Table 3:** Grade 4 Descriptive Statistics and Latent Profile Analysis Coefficient and Confidence Interval Results.

	All Participants (*n* = 155)		Cluster 1 (*n* = 9, 78% Girls)		Cluster 2 (*n* = 52, 37% Girls)		Cluster 3 (*n* = 6, 33% Girls)		Cluster 4 (*n* = 88, 65% Girls)	
Variable	M	SD	Coef.	95% CI	Coef.	95% CI	Coef.	95% CI	Coef.	95% CI
Motor skills	65.02	8.17	56.83	[52.74, 60.92]	71.91	[68.44, 75.39]	51.88	[46.82, 56.94]	62.45	[59.62, 65.28]
Accuracy	2.0 × 10^−4^	1.08	0.40	[−0.17, 0.96]	−0.77	[−1.10, −0.45]	2.21	[0.64, 3.78]	0.29	[−0.14, 0.72]
APR	2.07	0.82	1.24	[0.69, 1.79]	2.04	[1.78, 2.31]	2.11	[1.24, 2.98]	2.19	[1.98, 2.41]
With whom	3.18	0.61	2.27	[1.76, 2.77]	3.32	[3.10, 3.54]	3.64	[3.17, 4.11]	3.16	[2.97, 3.35]
Where	3.94	0.64	2.64	[2.20, 3.07]	4.07	[3.89, 4.25]	4.23	[3.76, 4.79]	3.99	[3.83, 4.15]

**Table 4:** Grade 5 Descriptive Statistics and Latent Profile Analysis Coefficient and Confidence Interval Results.

	All Participants (*n* = 155)		Cluster 1 (*n* = 17, 82% Girls)		Cluster 2 (*n* = 49, 53% Girls)		Cluster 3 (*n* = 4, 75% Girls)		Cluster 4 (*n* = 33, 52% Girls)		Cluster 5 (*n* = 23, 52% Girls)		Cluster 6 (*n* = 29, 45% Girls)	
Variable	M	SD	Coef.	95% CI	Coef.	95% CI	Coef.	95% CI	Coef.	95% CI	Coef.	95% CI	Coef.	95% CI
Motor skills	67.01	8.35	54.83	[51.21, 58.45]	74.34	[72.33, 76.35]	64.22	[58.83, 69.60]	61.99	[59.54, 64.45]	67.12	[64.05, 70.19]	68.09	[64.86, 71.32]
Accuracy	−9.0 × 10^−5^	1.21	1.34	[0.82, 1.87]	−0.79	[−1.10, −0.47]	−0.87	[−1.71, −0.03]	1.19	[0.83, 1.54]	−0.53	[−1.08, 0.01]	−0.29	[−0.7, 0.14]
APR	2.00	0.82	1.48	[1.15, 1.80]	2.48	[2.22, 2.73]	0.63	[−0.03, 1.29]	2.39	[2.11, 2.70]	1.41	[1.00, 1.82]	1.71	[1.41, 2.02]
With whom	3.23	0.60	2.85	[2.64, 3.05]	3.34	[3.17, 3.50]	2.09	[1.69, 2.49]	3.60	[3.45, 3.76]	3.74	[3.51, 3.97]	2.59	[2.38, 2.80]
Where	4.04	0.67	3.24	[2.98, 3.50]	4.07	[3.89, 4.24]	5.10	[4.50, 5.70]	4.25	[4.06, 4.45]	4.72	[4.44, 5.01]	3.50	[3.27, 3.73]

## Data Availability

Data supporting reported results can be obtained by contacting the contributing author.
